# SGLT2i, Canagliflozin mitigates amyloid‐beta pathology and cognitive decline in 5XFAD mice

**DOI:** 10.1002/alz70859_104072

**Published:** 2025-12-26

**Authors:** Dulmalika Navodya Herath Manchanayake, Hashan S. M. Jayarathne, Marianna Sadagurski

**Affiliations:** ^1^ Wayne State University, Detroit, MI USA

## Abstract

**Background:**

Aging is the primary risk factor for numerous chronic diseases, including neurodegenerative disorders such as Alzheimer’s disease (AD). Current therapeutic options for AD provide limited efficacy, thus requiring novel interventions that target key molecular pathways associated with aging and neurodegeneration. Pharmacological approaches targeting these pathways have shown promise in delaying aging and extending lifespan. A diabetes drug, Canagliflozin (Cana), an FDA‐approved sodium‐glucose cotransporter 2 (SGLT2) inhibitor, has previously demonstrated lifespan extension in genetically diverse UM‐HET3 male mice (14%) without effects in females. In aged male mice, Cana also exhibited neuroprotective properties, including improved central insulin responsiveness, reduced neuroinflammation, and enhanced locomotor activity and exploratory behavior. These results suggest that Cana has a potential for mitigating age‐associated brain pathologies.

**Method:**

To investigate this potential, we used a well‐established model of AD, 5XFAD mice and fed them diet containing Cana (180 ppm) from 3 months of age. At 6–7 months of age, we conducted metabolic assessments and a panel of behavioral assays, followed by brain histological analysis to assess amyloid‐beta (Aβ) burden, neuroinflammation, and hippocampal function.

**Result:**

Cana treatment significantly improved glucose tolerance in both male and female 5XFAD mice. However, only male 5XFAD mice exhibited notable improvements in cognitive performance, including enhanced spatial memory in behavioral assays. Importantly, Cana treatment markedly reduced hippocampal Aβ plaque burden and attenuated neuroinflammation in male 5XFAD mice.

**Conclusion:**

Our study highlights a novel role for SGLT2i in alleviating AD‐related pathologies. Moreover, for the first time, our findings emphasize SGLT2i as promising candidates for repurposing in the treatment of neurodegenerative diseases.

